# Visualizing nanoscale excitonic relaxation properties of disordered edges and grain boundaries in monolayer molybdenum disulfide

**DOI:** 10.1038/ncomms8993

**Published:** 2015-08-13

**Authors:** Wei Bao, Nicholas J. Borys, Changhyun Ko, Joonki Suh, Wen Fan, Andrew Thron, Yingjie Zhang, Alexander Buyanin, Jie Zhang, Stefano Cabrini, Paul D. Ashby, Alexander Weber-Bargioni, Sefaattin Tongay, Shaul Aloni, D. Frank Ogletree, Junqiao Wu, Miquel B. Salmeron, P. James Schuck

**Affiliations:** 1 Molecular Foundry, Lawrence Berkeley National Laboratory, 1 Cyclotron Road, Berkeley, California 94720, USA; 2 Materials Sciences Division, Lawrence Berkeley National Laboratory, 1 Cyclotron Road, Berkeley, California 94720, USA; 3 Department of Materials Science and Engineering, University of California Berkeley, 210 Hearst Mining Building, Berkeley, California 94720, USA; 4 Applied Science and Technology Graduate Program, University of California, 210 Hearst Mining Building, Berkeley, California 94720, USA; 5 Department of Chemistry, University of California Berkeley, 419 Latimer Hall, Berkeley, California 94720, USA; 6 Department of Materials Science and Engineering, Arizona State University, P.O. Box 876106, Tempe, Arizona 85287, USA

## Abstract

Two-dimensional monolayer transition metal dichalcogenide semiconductors are ideal building blocks for atomically thin, flexible optoelectronic and catalytic devices. Although challenging for two-dimensional systems, sub-diffraction optical microscopy provides a nanoscale material understanding that is vital for optimizing their optoelectronic properties. Here we use the ‘Campanile' nano-optical probe to spectroscopically image exciton recombination within monolayer MoS_2_ with sub-wavelength resolution (60 nm), at the length scale relevant to many critical optoelectronic processes. Synthetic monolayer MoS_2_ is found to be composed of two distinct optoelectronic regions: an interior, locally ordered but mesoscopically heterogeneous two-dimensional quantum well and an unexpected ∼300-nm wide, energetically disordered edge region. Further, grain boundaries are imaged with sufficient resolution to quantify local exciton-quenching phenomena, and complimentary nano-Auger microscopy reveals that the optically defective grain boundary and edge regions are sulfur deficient. The nanoscale structure–property relationships established here are critical for the interpretation of edge- and boundary-related phenomena and the development of next-generation two-dimensional optoelectronic devices.

## Abstract (web summary)

Understanding the dynamics of light-induced carriers is vital for employing two-dimensional materials in optoelectronic applications. Here, the authors use a sub diffraction-limit optical technique to reveal the excitonic properties of monolayer molybdenum disulfide at the nanoscale.

## Graphical abstract

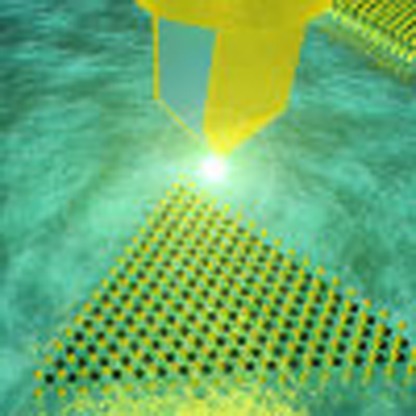

The emergence of two-dimensional (2D) monolayer transition metal dichalcogenides (ML-TMDC) as direct bandgap semiconductors^1^,^2^ has rapidly accelerated the advancement of room temperature, 2D optoelectronic devices^3^,^4^,^5^,^6^,^7^,^8^,^9^,^10^. However, performance of the active ML-TMDC materials often falls far below theoretical expectations, particularly for critical factors such as carrier mobility and quantum yield^4^,^11^. Overcoming such macroscopic limitations in lower-dimensional systems requires a nanoscale understanding of the materials. Central to 2D optoelectronic applications, light-matter interactions in ML-TMDCs are dominated by a manifold of tightly bound exciton states^12^,^13^,^14^ with remarkably strong absorption cross-sections^1^ and appreciable photoluminescence (PL)^2^. Unlike traditional 2D quantum wells, the enhanced coulombic interaction between the electrons and holes in ML-TMDCs stabilizes the excitonic states at room temperature. The most prevalent states are the low-energy A exciton and the charged A− trion^15^, and their relative populations can be tuned by electrostatically changing the electron density of the material^15^. Furthermore, adsorbates^16^, strain^17^,^18^ and piezoelectric^7^ effects can reversibly modify the energy of these states, whereas structural discontinuities such as grain boundaries^19^ (GBs) and defects^16^ can enhance or quench luminescence.^19^ While scanning tunnelling^20^,^21^ and transmission electron^19^,^22^,^23^ microscopies have probed atomic-scale electronic properties and structural defects in ML-TMDCs, optical investigations of these excited states have been diffraction limited and thus unable to directly resolve nanoscale excitonic phenomena. Near-field optical microscopy provides a route to explore material properties below the diffraction limit in 2D systems^24^,^25^,^26^,^27^. However, nanoscale optical visualization and spectroscopy of inelastic light-matter interactions (such as nano-PL) in two dimensions constitutes a formidable challenge, necessitating a non-traditional approach that confines optical excitation and collection without hindering spectral analysis.

Here we utilize the previously established sub-diffraction hyperspectral imaging capability of the Campanile probe^28^ to spectroscopically map nanoscale excited-state relaxation processes in chemical vapour deposition (CVD)-grown MoS_2_ on length scales that are commensurate with characteristic optoelectronic properties (for example, the exciton diffusion length and defect separations). The enhanced resolution reveals significant nanoscale optoelectronic heterogeneity and enables the quantification of exciton-quenching phenomena at GBs. Further, in-depth analysis of the nanoscale spatial irregularities of the excited-state PL reveals a disordered edge region that is ∼300-nm wide and has important implications for device engineering and edge-related phenomena.

## Results

### Nanoscale heterogeneity in the PL of ML-MoS_2_

**Figure 1:**
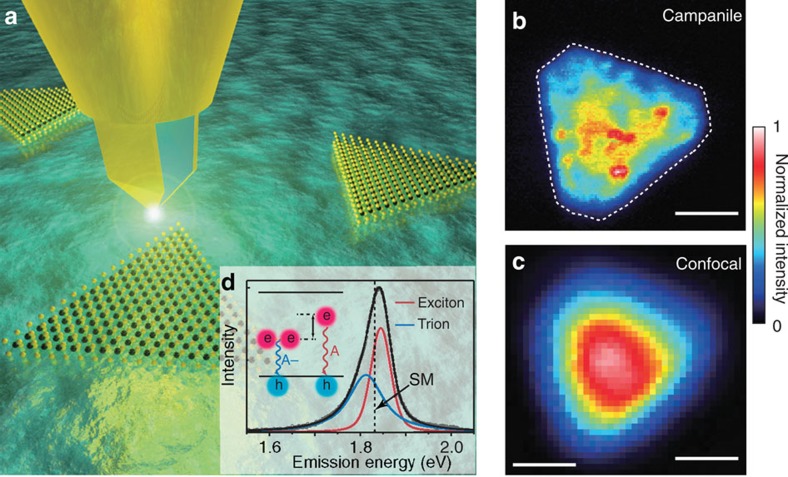
Nano-optical imaging of PL in ML-MoS_2_. (**a**) Illustration of near-field excitation and collection of the PL from ML-MoS_2_ using the Campanile near-field probe where the optical laser excitation (2.33 eV) and collection of sample emission are confined to the apex of the tip^28^ to produce high-resolution optical maps. At each pixel a full PL spectrum is acquired. (**b**) Map of the PL emission intensity of a triangular ML-MoS_2_ flake using the Campanile probe. The white dashed line indicates the flake boundary as determined from the shear-force topography. Scale bar, 1 μm. (**c**) An image of the same flake acquired with scanning confocal microscopy using a × 100, 0.7-NA air objective. Scale bar, 1 μm. (**d**) Near-field nano-PL spectrum averaged over the spatial extent of the ML-MoS_2_ flake. The emission contains two peaks arising from the radiative recombination from exciton (A) and trion (A−) states^15^. The dashed vertical line shows the position of the spectral median (SM) that splits the spectrum into equal amounts of high and low energy counts and is used to quantify spectral variations in the lower signal-to-noise spectra of individual spatial positions acquired during fast scans. All data were acquired under ambient conditions. NA, numerical aperture.

Compared with ML-TMDCs produced by exfoliation from bulk crystals, CVD-grown ML-TMDCs are appealing for device applications where high yield and large area production is required. Figure 1a illustrates hyperspectral near-field microscopy of CVD-grown ML-MoS_2_ (see Supplementary Fig. 1 and Supplementary Note 1 for optical characterization and synthesis details). With the Campanile probe, optical excitation and collection are spatially confined to the nanogap at the apex of the tip, which is scanned over the sample, recording a full emission spectrum at each position^28^ (see Supplementary Figs 2 and 3 and Supplementary Notes 2 and 3 for experimental and fabrication details). The spatial distribution of the integrated PL intensity (Fig. 1b) exhibits nanoscale fluctuations that are unresolved by conventional confocal optical microscopy of the same flake (Fig. 1c). Simultaneously collected topographic images indicate that these nanoscale variations are not topographical artefacts (Supplementary Fig. 4). Thus, significant nanoscale optoelectronic disorder is present in these materials.

Spectral analysis of the PL averaged over the extent of the flake reveals that the emission arises from the radiative recombination of the A exciton at ∼1.84 eV and its A− trion at ∼1.81 eV (ref. 15), which overlap strongly, as shown in Fig. 1d. The relative energies and populations of these two states intimately depend on numerous factors such as carrier and defect densities, screening, strain and band-bending effects^16^,^17^,^18^. Correspondingly, the nano-PL spectrum that is acquired at each point contains a wealth of information about the local optoelectronic properties. To robustly map spectral variations in the nano-PL, we characterize single-point emission spectra using the ‘spectral median', which is defined as the emission energy that divides the PL into low- and high-energy regions of equal intensity (Fig. 1d). While the spectral median in itself fails to deconvolve energetic shifts from changes in the relative intensities of the states, it more reliably captures relative spectral changes in noisy data than numerical fitting.

**Figure 2:**
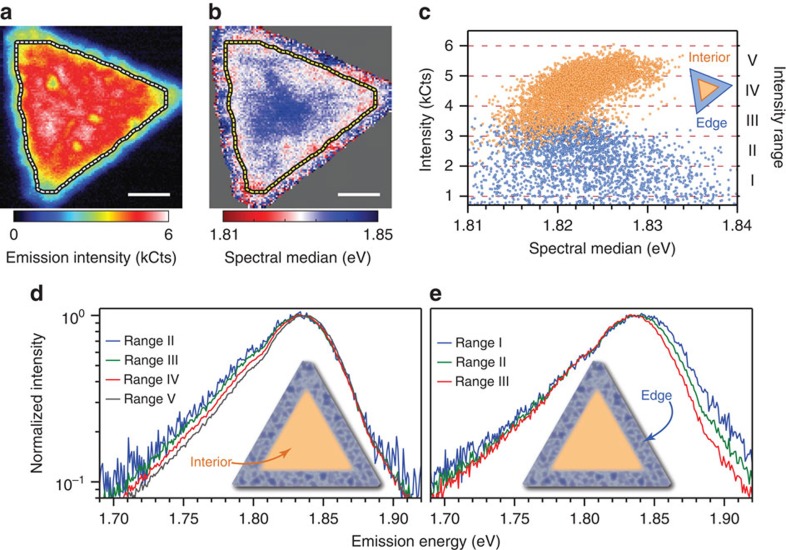
Optoelectronic discrimination between edge and interior regions of ML-MoS_2_. (**a**,**b**) Nano-PL images of emission intensity and SM (defined in Fig. 1d), respectively, of a single flake of ML-MoS_2_. The dotted line marks the boundary between the interior of the flake and a ∼300-nm wide periphery edge. Scale bars, 1 μm. (**c**) Emission intensity of each pixel plotted against its spectral median value for the interior (orange data points) and the edge regions (blue data points). Each data point in **c** corresponds to an emission spectrum recorded at a different position. The emission spectra from the interior and edges can be further grouped by their total emission intensity into one of five ranges (I, II, III, IV and V). (**d**) Averaged emission spectra of the interior data points for the intensity ranges II, III, IV and V (range I does not contain any interior data points) plotted on a normalized, semi-log scale for comparison. (**e**) Likewise, average emission spectra for the data points from the edge region for the intensity ranges I, II and III (ranges IV and V do not contain any data points from the edge region).

A nano-PL map of another ML-MoS_2_ flake is shown in Fig. 2. Spatial variations in the intensity (Fig. 2a) and emission energy (Fig. 2b) are observed across the flake in addition to a decrease in PL intensity near the edges. These trends are observed in all of the ML-MoS_2_ flakes that were investigated in this study (Supplementary Fig. 5) and do not change over an order of magnitude of excitation power (Supplementary Fig. 6). In ML-MoS_2_, increased relative emission of the trion is correlated with a reduction in the overall PL intensity^15^. To explore this type of behaviour in our spatially resolved data, we plot the integrated intensity of the PL acquired at each spatial position versus its emission energy (that is, its spectral median) in Fig. 2c. Two distinct clusters are observed. By systematically segregating the data points into either an internal interior region or a peripheral edge region (see Supplementary Fig. 7 for details), we find that the brighter cluster of correlated data points (orange data points) belongs to the interior of the ML-MoS_2_, while the significantly more scattered, dimmer cluster of points (blue data points) belongs to an ∼300-nm peripheral edge region. In the interior, the intensity and energy of the PL are correlated, with the higher-energy emission tending to be brightest. Such behaviour is consistent with increased emission intensity from regions with reduced trion populations. In contrast, the emission energy of the PL from the edge region is more disordered, spanning nearly the entire range of emission energies at the lower emission intensities.

As previously noted, shifts in the spectral median value can arise from changes in the relative amounts of exciton and trion emission and/or energetic shifts of the excitonic resonances. So, the correlation between the PL intensity and its spectral median alone cannot unambiguously unravel the underlying optoelectronic behaviour of the edge and interior regions. Thus, to further probe the behaviours observed in the correlation plot of Fig. 2c, the emission spectra are grouped by their total emission intensity into one of five equally spaced ranges spanning 1–6 kCts (ranges I, II, III, IV and V in Fig. 2c). For each intensity range, the emission spectra are summed, and the average spectrum is calculated as shown in Fig. 2d,e for the interior and edge regions, respectively (see Supplementary Fig. 8 for alternative intensity ranges). In this manner, the spectral characteristics of bright areas to those of dim areas can be compared with significantly improved signal-to-noise ratios over the single-pixel data. In the interior, we confirm that the primary spectral difference between bright and dim regions is the relative intensity of the low-energy emission from the trion state (Fig. 1d). Dim areas exhibit enhanced relative trion emission, indicating that the local PL intensity inversely correlates with the population of trions. This behaviour likely arises from localized regions of increased carrier density that enhance the formation of trions and increase non-radiative Auger recombination, which reduces the local PL quantum yield^15^.

Surprisingly, the PL of the edge region does not exhibit systematic variations in the relative intensity of the trion. Instead, PL emission from dimmer areas in the edge region (range I, Fig. 2e) is broadened to higher energies, indicating that the edge region is substantially more disordered than the interior. Further, emission from lower-energy states is apparently favoured in the edge region. Such a spectral signature implies the edge forms a 2D mosaic of localized, inhomogenously broadened emitters where excitations are funneled to low-energy sites similar to other mesoscopically disordered semiconductor systems such as organic conjugated polymers^29^ and quantum-dot solids^30^ (see Supplementary Note 4 for further discussion). It is clear that the edge region in synthetic MoS_2_ is more complicated than just its atomic termination and a metallic edge state^20^,^21^,^31^. Because such disorder is foreseeably deleterious for carrier transport, it may also shed light on carrier mobility in MoS_2_ where both hopping and band transport mechanisms have been reported^11^. Presumably, hopping mechanisms dominate in the disordered edge area as opposed to high mobility band transport in the interior. Future studies that combine nano-PL with scanning tunnelling microscopy (STM) or time-resolved spectroscopy will prove crucial in more precisely characterizing this critical edge region in ML-MoS_2_.

**Figure 3:**
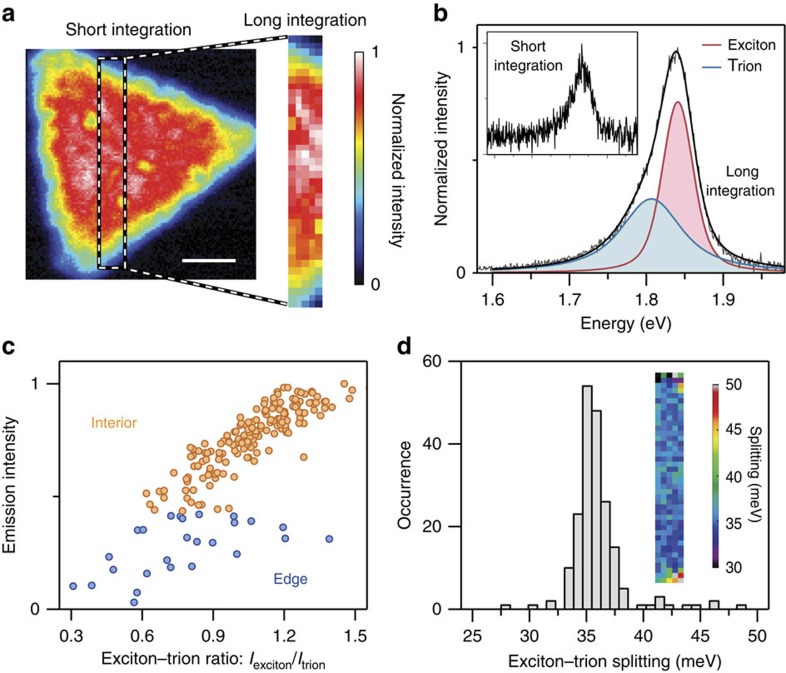
Imaging spatial variations of the relative population and energetics of the A− trion. (**a**) A smaller region (dashed rectangular box) of the ML-MoS_2_ flake from Fig. 2 is imaged with a longer pixel integration time (10 s) and reduced pixel density (100 nm per pixel) to increase the signal to noise of the near-field spectra. Scale bar, 1 μm. (**b**) Representative spectrum from a single pixel showing the improved signal-to-noise ratio of the 10 s pixel integration time of the slow scan compared with the 0.2 s pixel integration time of the fast scan (inset; covering the same spectral range as the main panel). The reduced noise of the slow scan enables fitting of the spectrum as a sum (black line) of peaks for the exciton state (A; red curve) and the trion state (A−; blue curve). The peak profiles are largely Lorentzian, but the best fits were achieved with a Voigt profile that convolves a pure Lorentzian with an underlying Gaussian distribution to account for inhomogeneous broadening effects. (**c**) PL emission intensity plotted against the exciton–trion ratio for each point in the interior (orange data points) and in the edge (blue data points) regions. (**d**) Statistical distribution and spatial distribution (inset) of the energetic separation between exciton and trion states.

To confirm the excitonic nature of the optoelectronic variations of the interior region seen in Fig. 2, the spatial interplay between excitons and trions is explicitly resolved in a smaller region (0.5 × 4 μm; Fig. 3a) of the same MoS_2_ flake in Fig. 3. Single-point spectra were recorded with improved signal to noise using a longer integration time, enabling reliable fitting of the exciton and trion peaks (Fig. 3b). The emission intensity of each position is plotted against the corresponding ratio of the exciton and trion intensities in Fig. 3c and confirms that increased trion formation correlates with reduced PL quantum yield in the interior of the flake. Further, Fig. 3d shows the distribution of the exciton–trion splitting, indicating an average value of ∼36 meV for this sample. The energetic splitting of the two states is directly related to the sum of the trion-binding energy and the Fermi energy in the system^15^. Although the splitting appears to be relatively uniform spatially, subtle variations could still possibly be uncovered with even higher resolution measurements in the future. Further, the spread of the values (∼3 meV) is similar to that observed by changing the Fermi energy via electrostatic gating^15^, envisaging studies that combine gating functionality with nano-PL characterization.

### Excited-state quenching at grain boundaries

**Figure 4:**
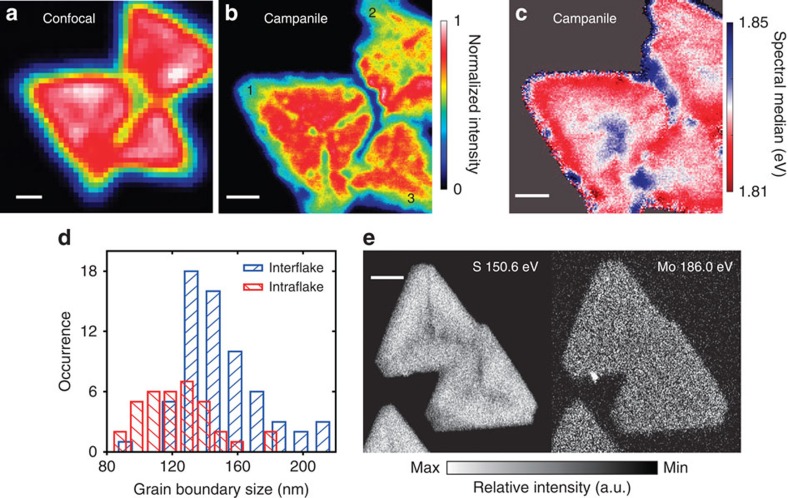
Excited-state quenching of GBs and elemental mapping of ML-MoS_2_. Far-field confocal micro-PL (**a**) and nano-PL (**b**) images of an aggregate of three flakes (labelled 1, 2 and 3) forming three interflake GBs. In the interior of flake 1, radial intraflake GBs are observed extending from the centre towards the apexes of the triangular flake. The interflake GB quenches the PL intensity by 50–80%, whereas the intraflake GB quenches the PL intensity by ∼20%. Scale bars, 1 μm. (**c**) Map of the emission energy (that is, the spectral median value defined in Fig. 1d). Scale bar, 1 μm. (**d**) Histograms of the half width at half max sizes of the interflake and intraflake GBs, which are measured from the spatial extent of the PL reduction and sampled semi-equidistantly along the respective features (see Supplementary Fig. 9 for more details). (**e**) Nano-Auger elemental mapping of S and Mo on a similar multiflake aggregate from the same growth run. Both the edge region and GBs are S-deficient, while the Mo composition is uniform over the flakes. Scale bar, 2 μm.

Whereas single-crystalline flakes of ML-MoS_2_ can be anatomized into an interior and edge, disruptions to the crystalline structure can also occur during the CVD growth process. Isolated polycrystalline MoS_2_ flakes can form intricate star-like structures while aggregates of flakes that merge during growth form complex polycrystalline patchworks^19^,^23^. The resulting GBs can significantly alter the local optoelectronic properties of the flake interior^19^,^32^. For example, some types of GBs are known to quench excitons, locally reducing the PL quantum yield^19^, but the limited resolution of conventional optical microscopy fails to precisely resolve the quenching phenomena. In Fig. 4, confocal micro-PL (Fig. 4a) and Campanile nano-PL (Fig. 4b) maps of three MoS_2_ flakes (labelled 1, 2 and 3) that merged during growth are compared. Although the confocal optical microscopy image (Fig. 4a) exhibits a mostly uniform PL intensity distribution within each flake, indicating a ‘high-quality' sample, substantial nanoscale fluctuations are observed in the nano-PL image. Furthermore, a reduction in the PL intensity that corresponds to an exciton-quenching region marks the boundaries between flakes 1 and 2 as well as flakes 2 and 3. This effect is better resolved by the Campanile probe, more precisely quantifying the reduction in PL intensity. In addition to these interflake GBs, three narrow regions where the PL is quenched by ∼20% extending radially from the centre of flake 1 (also in flake 3 and others shown in the Supplementary Fig. 5) are also better resolved in the nano-PL map. Interestingly, these GBs do not seem to alter the energetics of the PL, as can be observed in the nano-PL map of the emission energy in Fig. 4c, which is mostly devoid of systematic variations in the vicinity of the intra- and interflake GBs.

The structural width of a GB is on the order of the atomic-scale lattice spacing^19^,^23^, but acting as a non-radiative recombination centre^19^, its optoelectronic width (that is, the size of the quenching region) will be enlarged due to exciton diffusion (∼24 nm (ref. 19)), which itself can be locally enhanced by band bending or carrier-depletion zones. In Fig. 4d, the extents of the exciton-quenching regions are statistically quantified by analysing multiple line cuts across inter- and intraflake GBs (see Supplementary Fig. 9 for details). The widths show substantial disorder, varying between 80–160 and 90–210 nm for the intra- and interflake GBs, respectively, and are not limited by our estimated resolution of ∼60 nm (Supplementary Fig. 10 and Supplementary Note 5). Previous optical studies with limited resolution (∼500 nm) proposed that the exciton quenching at GBs could not be accounted for by exciton diffusion alone, but fell short of directly quantifying the effect^19^. Here the mesoscopic extent of exciton quenching is confirmed and quantified with average sizes of 150 and 120 nm for the inter- and intraflake GBs, respectively. Interestingly, nano-Auger spectroscopy maps of ML-MoS_2_ flakes from the same CVD growth run, shown in Fig. 4e (and Supplementary Fig. 11), reveal that the GBs and edge regions are S-deficient, consistent with previous results that show a ‘Mo-rich' edge^22^. Such S-deficient regions in MoS_2_ suggest probable *n*-doping near these boundaries^19^ and also are suspected to have a higher density of mid-gap trap states^19^,^22^, which may contribute to the local reduction in PL quantum yield.

## Discussion

The results that are presented here bridge STM investigations of the electronic structure of ML-MoS_2_ near GBs^32^ and edges^20^,^21^ to macroscopic optical imaging and spectroscopy measurements. In the vicinity of GBs, a complex reduction of the electronic bandgap over nanoscale distances is observed with STM^32^, which may also influence the exciton-quenching effect. Further, earlier STM work on ML-MoS_2_ has shown that an atomic metallic edge state is formed at the external boundary of the flakes^20^,^21^. Here we show that CVD-grown MoS_2_ flakes are also enclosed by a larger disordered edge region that we hypothesize forms during the termination of the growth process (that is, the cooling step)^22^. Thus, the edge of synthetic ML-MoS_2_ is more complex than just atomic-scale reconstructions of the crystalline interior—a crucial consideration that may help to explain observed edge-related phenomena such as resonantly enhanced second harmonic generation^31^ and even enhanced catalytic activity^33^. Ultimately, directly connecting these effects necessitates multimodal characterization using nano-PL techniques and STM on the same sample, which although challenging, is possible in principle and would be a very powerful study.

Altogether, our results provide a new insight into the rich optoelectronic properties of CVD-grown ML-MoS_2_ by resolving exciton relaxation processes with sub-diffraction resolution. Significant nanoscale heterogeneity is observed, and distinct optoelectronic systems corresponding to a pristine interior, disordered edges as well as intra- and interflake GBs in synthetic ML-MoS_2_ are revealed. Disorder induced by the growth process can produce a mesoscopic edge region that is more complex than just a reconstruction of the crystalline interior. This region must be considered when interpreting edge-related phenomena^31^,^33^ and perhaps avoided when making edge-specific electrical contacts to ML-MoS_2_ in optoelectronic devices^34^. Furthermore, the disordered edge region and GBs are expected to reduce device performance, especially carrier mobility and recombination efficiency. GBs are found to quench excitons over ∼150 nm, providing an initial quantitative basis to estimate their effects in 2D optoelectronic devices. Such detailed information on the subtle variations in optoelectronic properties and their relationship to corresponding nanometre scale structure within synthetic ML-TMDCs enhances the understanding of these materials, sets the stage for future multimodal nanoscale optoelectronic studies and will hopefully guide the future development of high-quality 2D materials and next-generation devices.

## Methods

### Sample growth

ML-MoS_2_ was grown on 100-nm SiO_2_/Si substrates via standard CVD growth procedures with MoO_3_ and S precursors at 700 °C under continuous flow of N_2_. See Supplementary Note 1 for a detailed description of the growth process and Supplementary Fig. 1 for optical and Raman characterization.

### Near-field and confocal PL imaging

Scanning near-field PL imaging was performed on a customized NT-MDT scanning near-field optical microscope in a shear-force configuration as fully described in Supplementary Note 2. The Campanile tips were scanned over the sample at a distance of ∼5 nm. The excitation source was a linearly polarized 532-nm continuous wave laser. We estimate that 4 μW of excitation power were launched into the Campanile structure. The PL emission was collected by the same Campanile structure and analysed with a spectrometer with a cooled charge-coupled device camera. The far-field imaging of the PL was performed on the same NTMDT microscope in an upright confocal configuration with a × 100, 0.7-numerical aperture objective using the same 532-nm excitation source at 4.3 μW as measured at the back of the objective. The emission was collected and analysed by the same experimental setup as in the near-field measurements.

### Campanile tip fabrication

The Campanile tip structures were fabricated at the end of an etched single-mode optical fibre as described in full detail in Supplementary Note 3. Scanning electron micrographs of the fabricated Campanile geometry are provided in Supplementary Fig. 3.

### Nano-Auger microscopy

Nano-Auger electron spectroscopy was performed on an Oxford/Omnicron Nano-Auger system under ultra-high vacuum of 10^−10^ mbar. The size of the electron beam spot was ∼10 nm. The typical uncertainty in the nano-Auger composition measurement is ∼3%.

## Additional information

**How to cite this article:** Bao, W. *et al.* Visualizing nanoscale excitonic relaxation properties of disordered edges and grain boundaries in monolayer molybdenum disulfide. *Nat. Commun.* 6:7993 doi: 10.1038/ncomms8993 (2015).

## Supplementary Material

Supplementary Information Supplementary Figures 1-11, Supplementary Notes 1-5 and Supplementary References
