# Supplementary material for: Visualizing nanoscale excitonic relaxation properties of disordered edges and grain boundaries in monolayer molybdenum disulfide
Source: Nat Commun. 2015 Aug 13;6:7993. doi: 10.1038/ncomms8993 (PMC4557266; doi:10.1038/ncomms8993)
Supplement: Supplementary Information — Supplementary Figures 1-11, Supplementary Notes 1-5 and Supplementary References [file ncomms8993-s1.pdf]

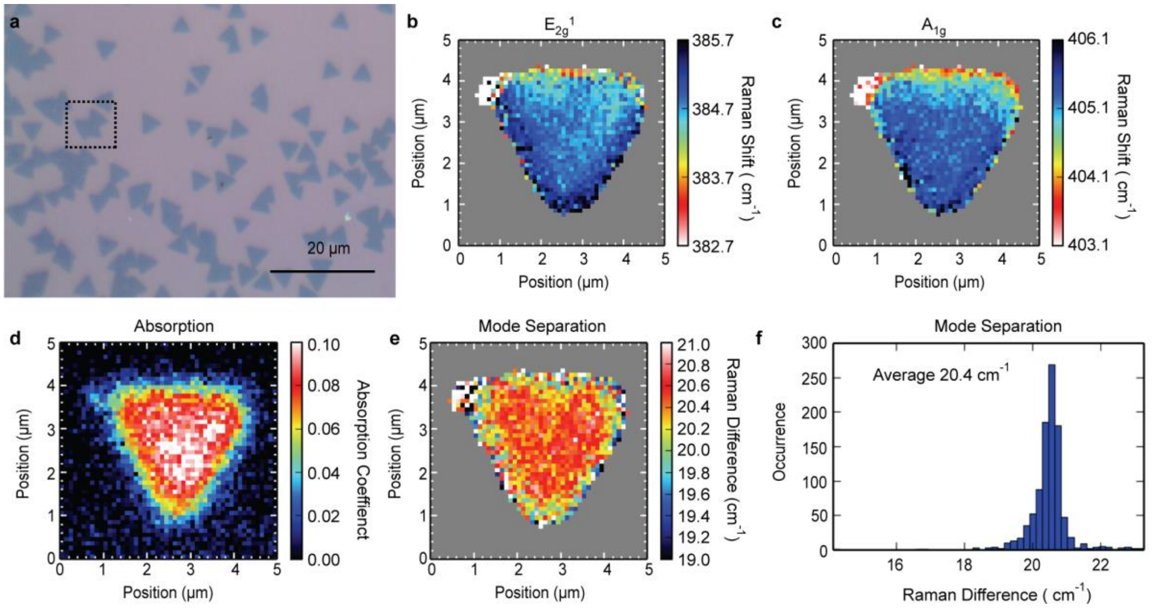


**Supplementary Figure 1** – Optical characterization of the $MoS_2$ sample used in these measurements. **a.** Bright field optical microscopy of the monolayers of $MoS_2$ on the $SiO_2$/Si growth substrate. The black dashed line marks the flake that is shown in Fig. 4 of the main text. **b, c.** Spatial maps of energies of the $E^1_{2g}$ and $A_{1g}$ modes measured using confocal Raman spectroscopy. **d.** Estimated $MoS_2$ absorption based on the relative attenuation of the Raman signal from the underlying Si substrate. **e, f.** The spatial and statistical distributions of the energetic splitting between the $A_{1g}$ and $E^1_{2g}$ vibrational modes.

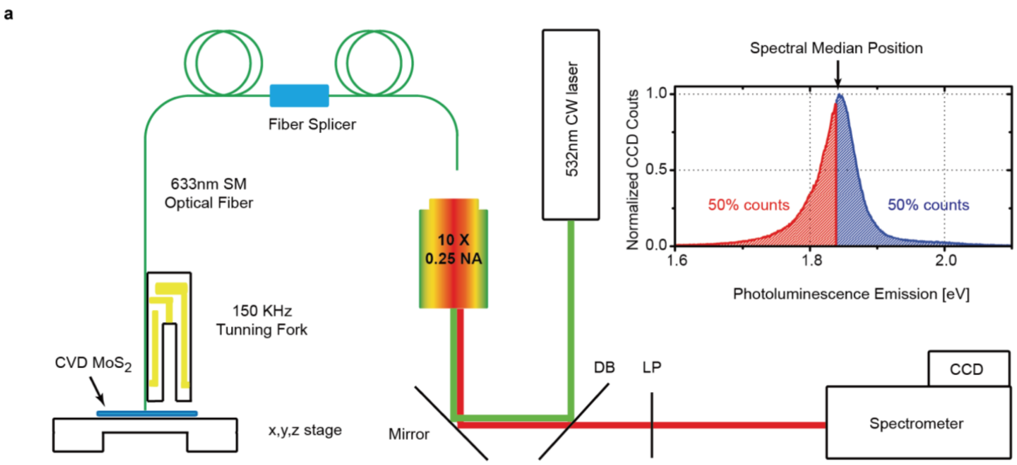


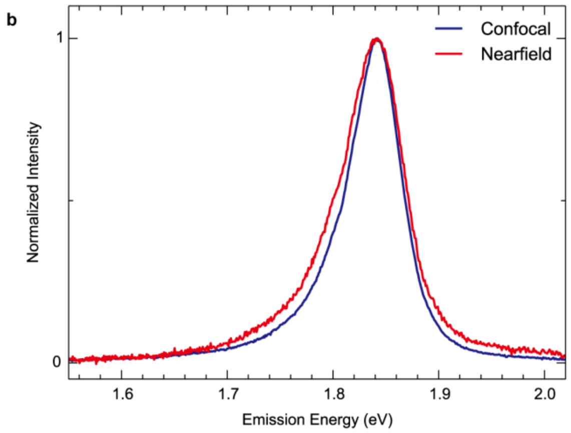


**Supplmentary Figure 2** – Nearfield hyperspectral optical microscopy with the Campanile probe. **a.** Schematic drawing of our optical beam path. **b.** A comparison of the spatially averaged emission spectra collected from the Campanile nearfield probe and traditional confocal microscopy (100× 0.7 NA objective) for the ML-$MoS_2$ flake presented in Figure 1. For the nearfield measurement, the laser power before the fiber-slicer was 4 µW. The same excitation power was used for the confocal microscopy measurement as measured at the back aperture of the objective. The average emission spectra of the confocal and nearfield datasets are qualitatively similar, exhibiting emission from the exciton and trion states. We note that the width of the PL spectrum from the nearfield dataset is slightly broader and could be indicative of a larger excitation rate at the apex of the tip.

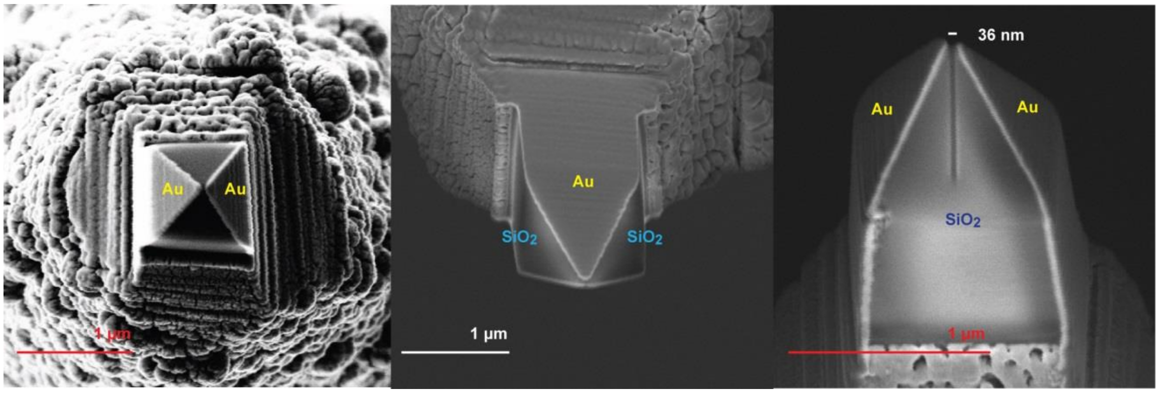


**Supplementary Figure 3 –** SEM images of a typical Campanile probe used in our measurement.

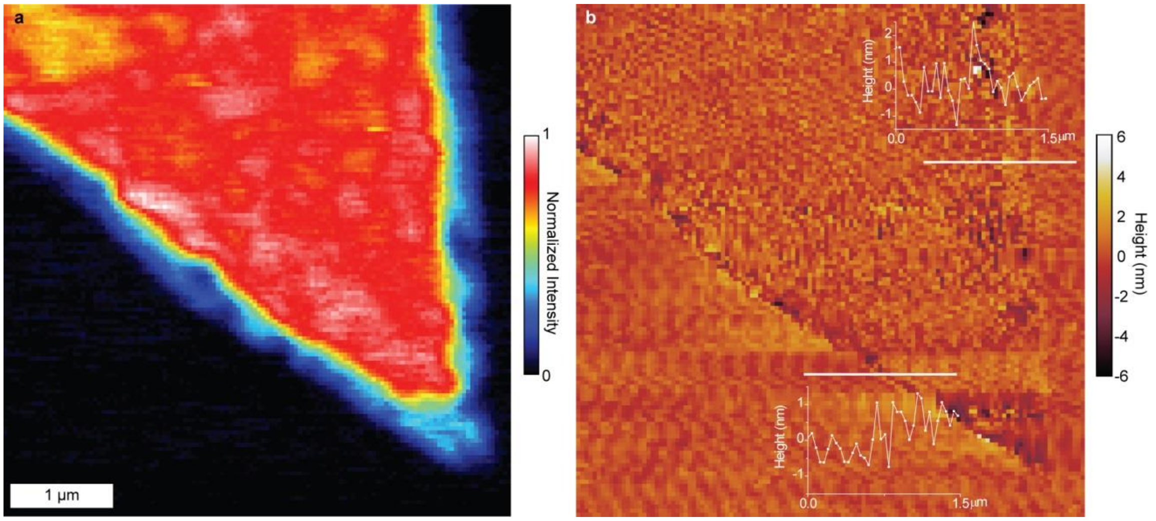


**Supplementary Figure 4 –** Comparison of a nano-PL map with the topography of a monolayer of $MoS_2$. **a.** A nano-PL map with nanoscale spatial variations. **b.** The measured topography from the shear-force feedback algorithm indicates that the monolayer of $MoS_2$ does not have topological features that correlate with the features seen in the PL. Two line cuts of its topography profile are also shown. The electronics of our shear-force microscope (NTMDT) typically exhibits a 0.5 nm noise in the height measurement. Since the physical size of the tip is larger than the nano-gap that determines the excitation volume, there is typically a 60 – 80 nm shift between the topography and optical datasets. The size of this shift is consistent with the thickness of the gold film on the Campanile probe.

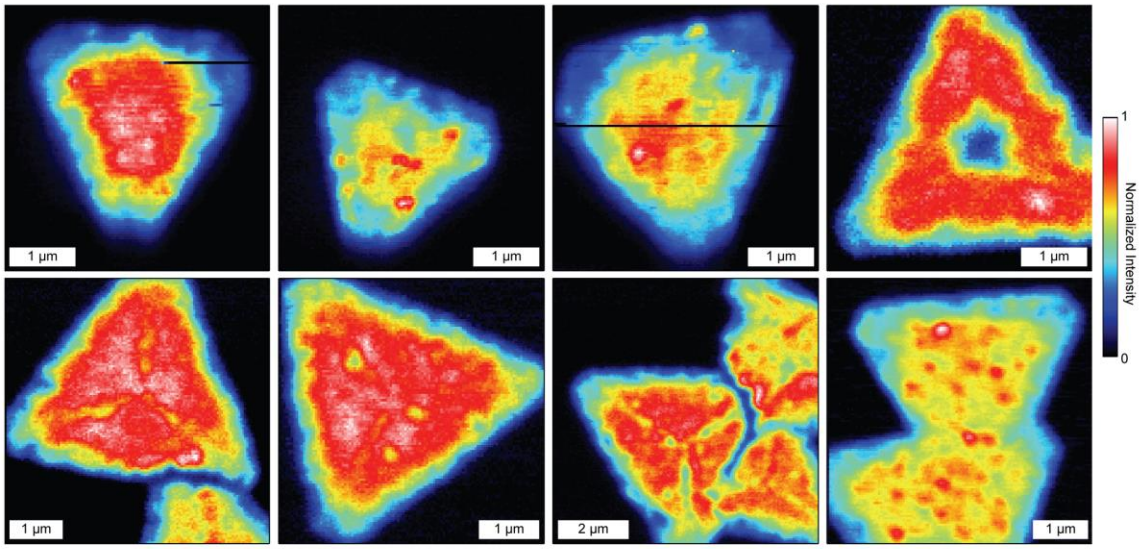


**Supplementary Figure 5 –** A collection of nano-PL maps from different monolayer $MoS_2$ flakes that were acquired during this study. All data were taken at the same excitation power as in the manuscript except for the upper-right panel where the excitation intensity was attenuated by a factor of 10.

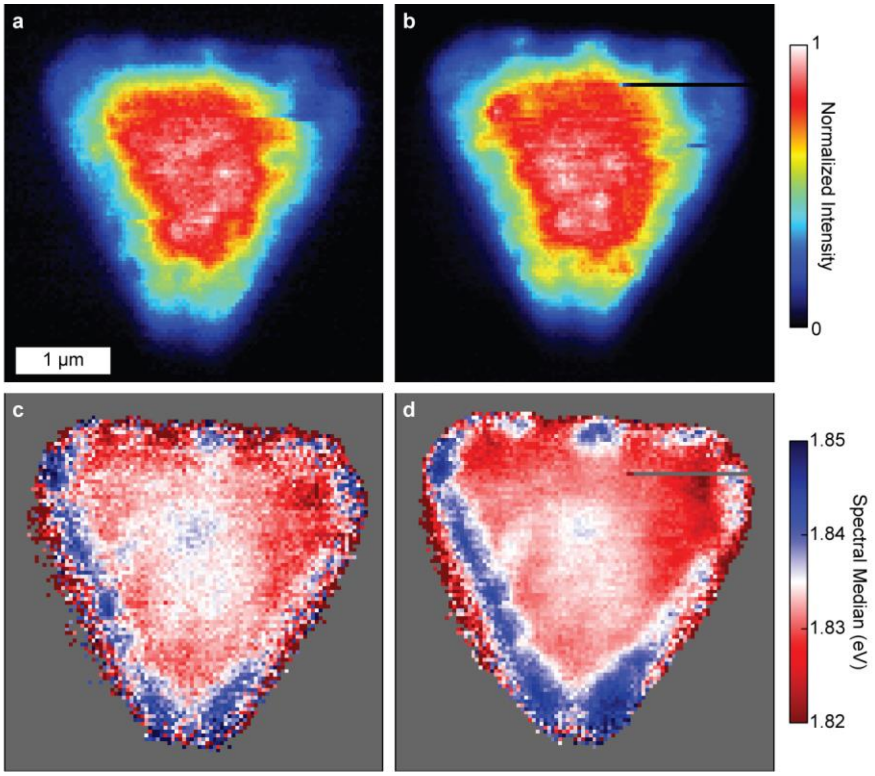


**Supplementary Figure 6** – Nano-PL mapping of monolayer $MoS_2$ at two different excitation powers. Panels **a** and **b** show the PL intensity at ~0.27 μW and 2.7 μW, respectively. Features that are observed at lower excitation intensity are also observed at higher intensities with an improved signal to noise ratio. Correspondingly, the maps of the spectral median at the lower power (panel **c**) and higher power (panel **d**) exhibit the same features and numerical values indicating robustness over an order-of-magnitude in excitation intensity.

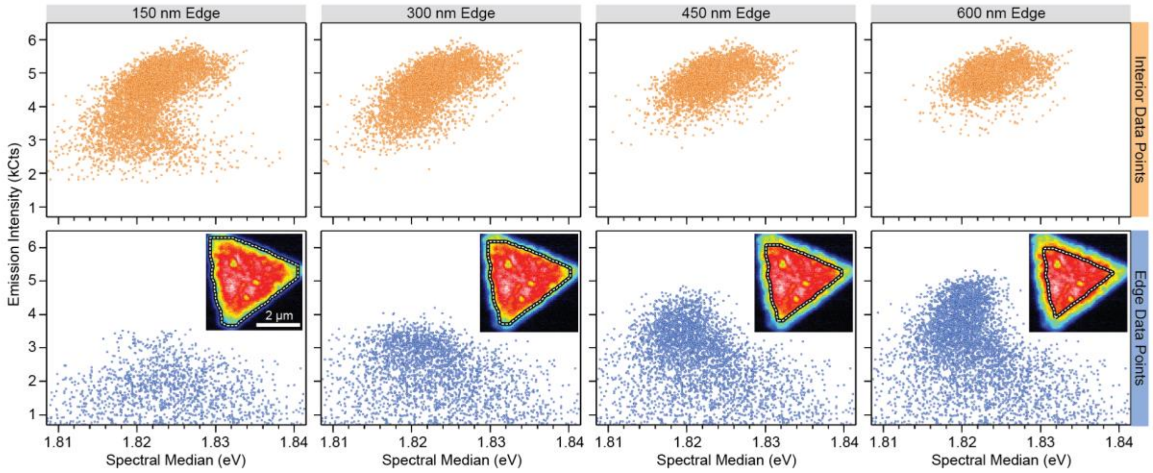


**Supplementary Figure 7 –** Emission intensity and spectral median correlation analysis for different edge widths. Each column in the above figure corresponds to the data analysis presented in Fig. 2c of the main text for a different edge width. The top row contains data points that are in the interior region of the flake, and edge points are in the bottom row. At an edge width of 150 nm (the first column), the correlation plot for the interior point exhibits two distinct features: a linear correlation and an uncorrelated band of points at low intensity. At edges widths of 450 nm and 600 nm (third and fourth column, respectively), two similar features emerges for the edge points. At an edge width of 300 nm (second column), the correlation plots for the interior and edges both exhibit a singular correlation.

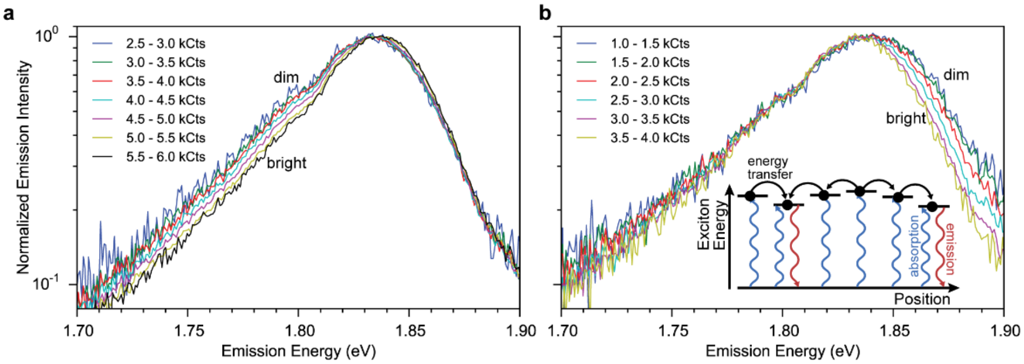


**Supplementary Figure 8** – Average spectra of the PL of the ML-$MoS_2$ flake from Figure 2 grouped into finer ranges of intensity for the edge and interior regions. **a.** From the interior region, the average emission spectra of individual points grouped into seven equally spaced ranges of total intensity spanning 2.5 – 6.0 kCts confirms the trend in Fig. 2d where dimmer PL contains a larger relative amount of low-energy emission from the trion state. **b.** From the edge region, the average emission spectra of individual data points grouped into six evenly spaced bins spanning 1.0 - 4.0 kCts confirms the trend in Fig. 2e of the main text where dimmer PL exhibits increased broadening of the main exciton peak to higher energies. Inset: a schematic depiction of energetically disordered localized states. Efficient coupling between the states (i.e., energy transfer processes) can funnel excitation energy to the lowest energy sites which then dominate the PL process**.**

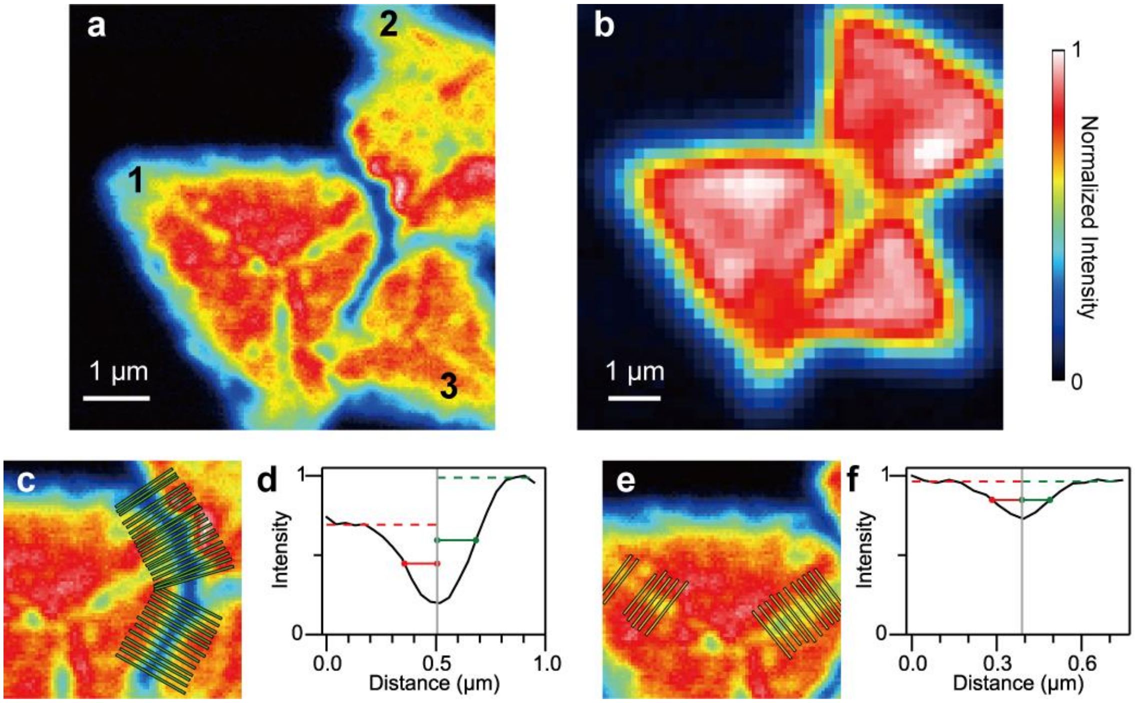


**Supplementary Figure 9** – Excited state quenching at interflake and intraflake grain boundaries. **a.** PL intensity map of a multiflake aggregate of three ML-$MoS_2$ flakes (labeled 1, 2 and 3 in black lettering) forming three interflake grain boundaries. In the interior of flake 1, intraflake grain boundaries are observed extending from the center towards the apexes of the triangular flake. The interflake boundary quenches the PL intensity by 50-80%, whereas the intraflake boundary quenches the PL intensity by ~20%. **b.** An image of the same flakes acquired with a traditional scanning confocal microscope using a 100×, 0.7 NA objective. The significant nanoscale heterogeneity of the PL (panel a) is masked by the non-local excitation and collection of far-field confocal optics, and the PL quenching by the grain boundary is under estimated[6]. **c.** The positions of the line-sections (green markers) where the spatial extent of exciton quenching by the interflake grain boundaries presented in Figure 4 were measured. **d.** A sample line-section of the reduction in PL across the interflake grain boundary and the measurement of the asymmetric half-width-at-half-maximum (red and green lines) from the position that corresponds to the minimum emission intensity (gray line). **e,f.** The same plots as c and d for the intraflake grain boundaries.

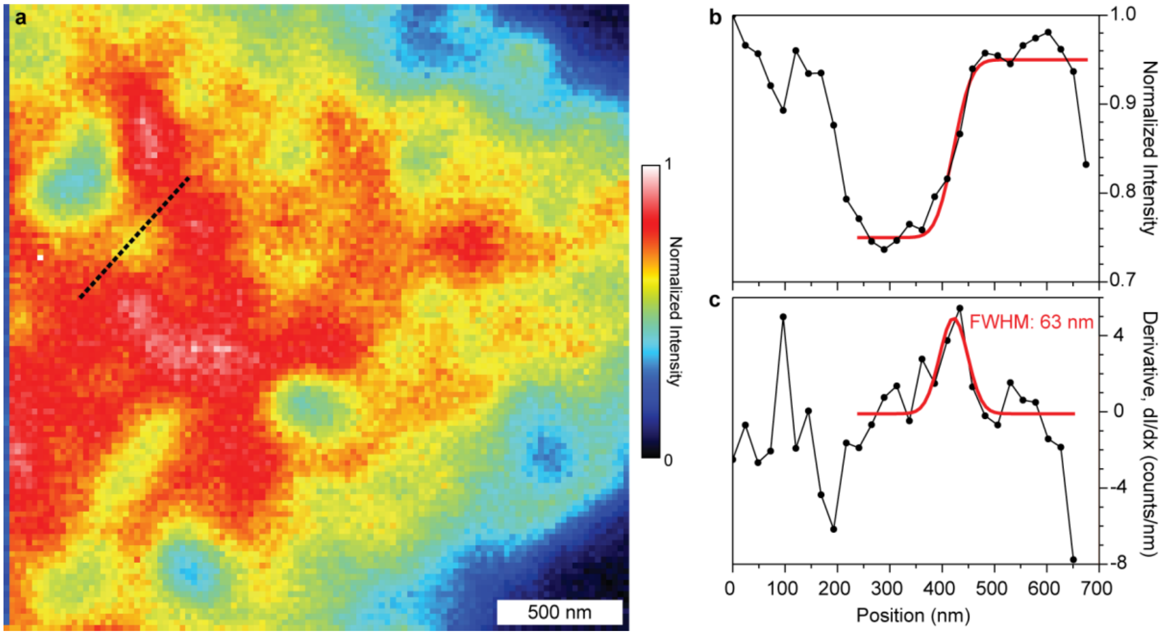


**Supplementary Figure 10** – Estimate of spatial resolution. **a.** Map of the PL intensity of a monolayer of $MoS_2$. The dashed line marks a cross section where a sharp transition from dim to bright emission is observed. The intensity interpolated along this line is shown in panel **b** along with its spatial derivative in panel **c**, where the red lines in both panels show the Gaussian peak that was fitted to the derivative and then integrated to produce a step-like feature in the intensity plot.

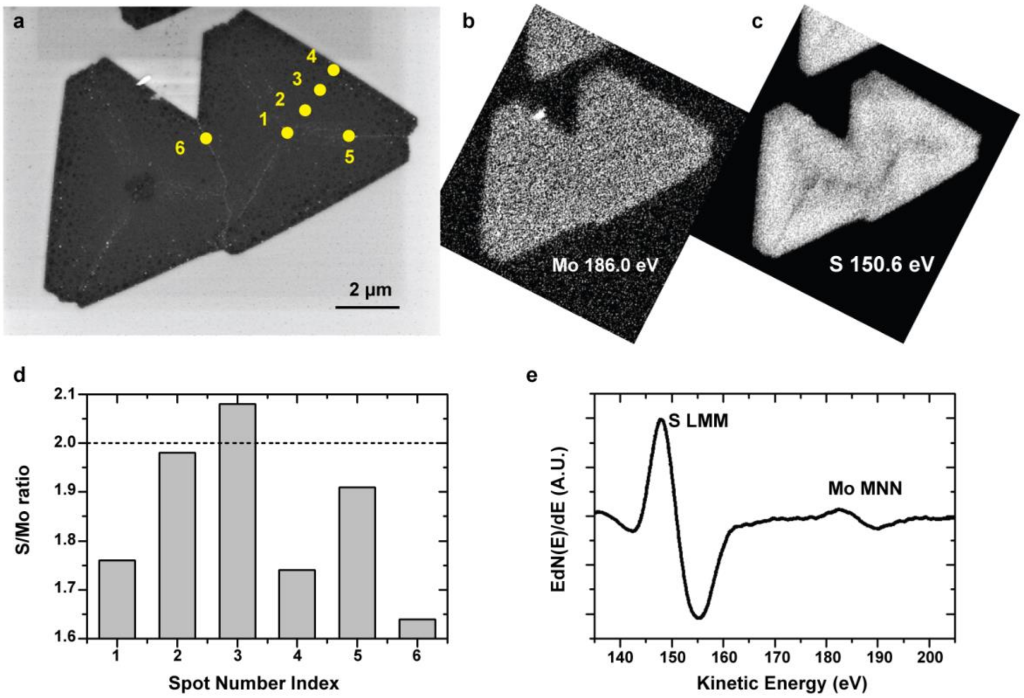


**Supplementary Figure 11** – Scanning Auger Microscopy Characterization of $MoS_2$ flakes. **a.** SEM image of the $MoS_2$ flakes used in nano-Auger experiment. **b,c.** Elemental mapping of Mo (panel b) and S (panel c) based on Mo MNN (186.0 eV) and S LMM (150.6 eV) Auger transitions. Both the edge and grain boundaries are S-deficient while Mo composition is almost uniform over the CVD flakes. However, in the case of flakes with no clear boundary structures, chemical inhomogeneity is not observed. **d.** The stoichiometric S/Mo ratio at different location was determined by the peak-to-peak heights of primary S LMM and Mo MNN transitions obtained from the first derivative Auger spectra[8]. Boundaries (spot 5 and 6), edges (spot 4) and flake nucleation centers (spot 1) were confirmed to be S-deficient. The cleaved surfaces of bulk $MoS_2$ single crystals (supplied from 2D semiconductor and SPI) were used as references for chemical composition analysis. **e.** A typical Auger spectra of the $MoS_2$ showing both the Mo and S transition peaks.

# Supplementary Note 1

## CVD monolayer $MoS_2$ sample preparation and characterization

Monolayers of $MoS_2$ were grown on 100 nm $SiO_2$/Si substrates via CVD. Prior to growth, the substrates were cleaned in Piranha solution for 2 hours and then washed with deionized (DI) water. The substrates were placed face-down on the top of an alumina crucible that contained 3 mg of $MoO_3$ powder. A second crucible with S was placed upstream ~19 cm away from the $MoO_3$ source. The system was purged with ultrahigh purity $N_2$ gas at a flow rate of 500 sccm for 10 min and then heated to 300 °C over 10 min with the $N_2$ flowing at 100 sccm. The system was then heated to 700 °C within 15 min under 5 sccm of $N_2$ flow and then held at these conditions for 3 min. The furnace was then powered off. When the temperature reached 680 °C, the furnace was slightly opened by inserting a small metal part to prop open the top lid. At a temperature of 550 °C, the furnace was completely opened and the growth tube was fully removed from the furnace to achieve rapid cooling of the growth reaction. Sulfur boils at 450 °C and in our configuration, that S vapor was delivered to the growth region at a flow rate of ~2 sccm during the growth. During the cooling stage, S is continually supplied to the sample at flow rates ranging from 2-5 sccm to prevent sample degradation. The samples were characterized with Raman spectroscopy and show a <20.5 $cm^{-1}$ separation between the $A_{1g}$ and $E^1_{2g}$ vibrational modes that is consistent with typical monolayer $MoS_2$ grown via CVD[1-3].

## Supplementary Note 2

## Experimental details of near-field imaging and spectroscopy

The near-field measurements were done on a customized NTMDT scanning nearfield optical microscope (NT-MDT NTEGRA Spectra) using a shear force head as shown in Supplementary Figure 2. An optical fiber that is terminated with the Campanile tip was glued to a tuning fork (2 mm × 6 mm; SCTF Electronics) with a resonance frequency of ~150 kHz. After attachment of the Campanile probe and in ambient conditions, a Q-factor of ~300 for the vibrational resonance was typically observed. The Campanile tip was kept ~5 nm above the sample during scanning, using the phase of the tuning fork oscillations for feedback (set point of 0.5°). Due to the temperature and pressure fluctuations in the lab, the tip was retracted 500 nm from the surface and re-approached after each line across the fast axis of the scan. While the tip was disengaged from the surface, the feedback loop was reset in order to account for temporal drift in the phase and maintain the stability of the tip-sample interaction over the full period of the measurement (2-8 hours). Linearly polarized excitation at 532 nm (Coherent Inc. Sapphire SF 532-100 CDRH) was spectrally filtered with a narrowband filter and coupled with an efficiency of 60% into the core of a patch single-mode optical fiber. At the exit of this patch fiber, the power of the excitation laser was ~4 μW. A mechanical fiber splicer (Thorlabs TS125) with a coupling efficiency about ~70% was used to connect the patch fiber to the fiber with the Campanile tip. Thus, we estimate that ~2.7 μW is launched into Campanile structure. The orientation of the polarization of the excitation light inside the fiber is controlled with an in-line Babinet–Soleil fiber polarization controller (Newport

Inc. F-POL-IL). The $MoS_2$ PL signal was collected with the same Campanile tip, optical fibers and coupling assembly. Two long-pass filters were used to filter the excitation light from the sample emission, which was then imaged onto a 200 μm circular entrance aperture of a spectrometer and ultimately detected with a cooled CCD camera (Andor iDus CCD DV401A-BV-600). For large area scans, hyperspectral maps were recorded with 200 ms integration time for each pixel, whereas the data in the smaller region (Fig. 3a) used a 10 s integration time.

The confocal μPL measurements were also performed with the same NT-MDT setup. A 100×, 0.7 NA objective was used to excite the sample at 532 nm and collect the resulting photoluminescence or Raman signal. The emission was analyzed through the same spectroscopy setup, employing a 50 μm pinhole.

## Supplementary Note 3

## Fabrication details for the Campanile probe

For a single Campanile tip, a Nufern S630-HP pure silica core single mode optical fiber (3.5 μm core diameter) was wet etched with 40% HF to shape a smooth cone with a half-cone-angle of ~18° and a tip-radius that is less than 200 nm. A 300 nm thick layer of a mixture of Pt and Au was sputtered on the fiber surface to ensure high conductivity in $Ga^+$ focused ion beam (FIB) milling (Zeiss Crossbeam 1540) process. The preliminary Campanile geometry was carved into the etched glass fiber tip by FIB milling. A subsequent 300 nm Pt/Au layer was deposited via sputtering to recoat the newly exposed $SiO_2$ surfaces with a conductive metal. A second FIB milling process was used to expose two opposing sides of the Campanile structure. Using tilt evaporation at 24°, a 3 nm Ti adhesion layer followed by a 70 nm thick Au layer and finally a 20 nm thick Cr protection layer were evaporated onto the structure on the $SiO_2$ surfaces that were exposed in the second FIB milling step to form the opposing metallic plates of the Campanile structure. After a final milling step to refine the shape and ensure that the metal plates are not connected, Cr etchant was used to strip the superfluous Cr layers, and finally a 2 nm thick layer of $Al_2O_3$ was deposited on the structure by atomic layer deposition at 40 °C. SEM micrographs of the final Campanile structures are shown in Supplementary Figure 3. Typically the resolution of the angle resolved evaporation was not sufficient to create a well-defined gap between the two triangular metallic plates at the apex. Thus, the gap was further cut using FIB milling, yielding gaps with dimensions that were typically 35-40 nm wide and 50 nm long (Supplementary Figure 3).

## Supplementary Note 4

## Intensity-spectrum correlation in the spatial variations of photoluminescence from monolayer $MoS_2$

In Figure 2c of the main text, it is shown that the spatial variations of the photoluminescence spectrum are correlated with variations in the integrated emission intensity. Further, the correlated behavior in the interior is distinct to that of a 300 nm wide peripheral edge region. To unravel the origins of the spectral fluctuations, we calculate the average emission spectra of sets of the individual hyperspectral data points that are grouped in ranges of increasing total emission intensity in Figures 2d and 2e for the interior and edge regions, respectively. We use the non-normalized spectrum (i.e., raw CCD counts) of each data point in the group to compute the average emission spectrum at the particular intensity level. This methodology enables the comparison of the typical emission spectrum of the dim regions of the $MoS_2$ flake to that of the bright regions. In Figs. 2d and 2e in the main text, we performed this analysis by dividing the data points into five intensity ranges that span an interval of 1000 counts (ranges I – V). In Supplementary Figure 8, the same analysis is repeated on the data from Figure 2 using smaller bin sizes (500 counts) to demonstrate that the identified trends are not artefacts of the choice of binning.

For the interior region (Figs. 2d and Supplementary Figure 8), the emission from dim regions of $MoS_2$ compared with that of the bright regions is found to have more relative low energy emission from the trion state with respect to the main exciton peak. In the edge region (Figure 2e and Supplementary Figure 8b), the behavior is markedly different.

Almost no variation is seen in the relative amount of trions in the PL, and the emission from the dim regions of the peripheral edge exhibits a high-energy broadening of the main exciton peak (an effect that is absent in the interior region). As discussed in the main text and depicted in the inset of Supplementary Figure 8b, such behavior is reminiscent of a disordered system that is comprised of an ensemble of localized states of different energies that are efficiently coupled, such as the chromophoric units of conjugated polymers[4] or individual quantum dots in a quantum dot solid[5]. Although all of states can be efficiently optically excited, efficient coupling between the states (i.e., energy transfer) rapidly depopulates the higher-energy states and funnels the excitation energy into the lowest energy sites which in turn dominate radiative relaxation (i.e., PL). This extra relaxation mechanism of the high-energy sites effectively reduces their PL quantum yield, thus reducing their brightness. Time-resolved spectroscopy combined with the nano-PL technique that is presented here could shed additional light on this relaxation mechanism. Because the higher-energy sites are predicted to have an additional, efficient non-radiative relaxation mechanism, their excited state lifetime should be substantially shorter than that of the lower-energy sites which lack this additional non-radiative relaxation pathway. Thus, spectrally and even possibly spatially resolved transient PL spectroscopy could provide significant insight into the optoelectronics of this peculiar edge region.

## Supplementary Note 5

## Estimate of spatial resolution

Without a single point emitter or discrete edge in the monolayer $MoS_2$, the spatial resolution can only be estimated from the sharpest observed features, which ranged from 50-80 nm. In Supplementary Figure 10, one such feature is extracted from the map of the PL intensity (Supplementary Figure 10a). Here, the feature corresponds to the transition from a dim area to a bright area (Supplementary Figure 10b), which can be analyzed using the knife-edge resolution test[7] to estimate our spatial resolution. Because the feature is a step function, its spatial derivative (Supplementary Figure 10c) is well-described by a Gaussian peak with a width that directly maps to the width of the transition region[7]. The full-width-at-half-maximum of the peak in the spatial derivative is 63 nm, corresponding to a $2\sigma$ width of 54 nm. In terms of a step (Supplementary Figure 10b), the $2\sigma$ width marks the extent of the transition from 15% to 84% of the step height, providing an estimate of our spatial resolution.

## Supplementary References


1 Liu, K. *et al.* Elastic properties of chemical-vapor-deposited monolayer $MoS_2$, $WS_2$, and their bilayer heterostructures. *Nano Lett.* **14**, 5097-5103 (2014).

2 Najmaei, S. *et al.* Vapour phase growth and grain boundary structure of molybdenum disulphide atomic layers. *Nature Mater.* **12**, 754-759 (2013).

3 Liu, K. H. *et al.* Evolution of interlayer coupling in twisted molybdenum disulfide bilayers. *Nature Commun.* **5**, 4966 (2014).

4 Laquai, F., Park, Y. S., Kim, J. J. & Basche, T. Excitation Energy Transfer in Organic Materials: From Fundamentals to Optoelectronic Devices. *Macromol. Rapid Commun.* **30**, 1203-1231 (2009).

5 Kagan, C. R., Murray, C. B., Nirmal, M. & Bawendi, M. G. Electronic Energy Transfer in CdSe Quantum Dot Solids. *Phys. Rev. Lett.* **76**, 1517-1520 (1996).

6 van der Zande, A. M. *et al.* Grains and grain boundaries in highly crystalline monolayer molybdenum disulphide. *Nature Mater.* **12**, 554-561 (2013).

7 Levenson, E., Lerch, P. & Martin, M. C. Spatial resolution limits for synchrotron-based spectromicroscopy in the mid- and near-infrared. *Journal of Synchrotron Radiation* **15**, 323-328 (2008).

8 Childs, K. D. *et al.* Handbook of Auger electron spectroscopy. *Physical Electronics* (1995).
